# Genetic variation in the 3′-UTR of *CYP1A2, CYP2B6, CYP2D6, CYP3A4, NR1I2*, and *UGT2B7*: potential effects on regulation by microRNA and pharmacogenomics relevance

**DOI:** 10.3389/fgene.2014.00167

**Published:** 2014-06-04

**Authors:** Marelize Swart, Collet Dandara

**Affiliations:** Pharmacogenetics and Cancer Research Group, Division of Human Genetics, Department of Clinical Laboratory Sciences, Faculty of Health Sciences, University of Cape TownCape Town, South Africa

**Keywords:** bioinformatics prediction, drug metabolizing enzymes, microRNA, miRSNPs, pharmacogenomics, polymorphisms

## Abstract

**Introduction:** Pharmacogenomics research has concentrated on variation in genes coding for drug metabolizing enzymes, transporters and nuclear receptors. However, variation affecting microRNA could also play a role in drug response. This project set out to investigate potential microRNA target sites in 11 genes and the extent of variation in the 3′-UTR of six selected genes; *CYP1A2, CYP2B6, CYP2D6, CYP3A4, NR1I2*, and *UGT2B7*.

**Methods:** Fifteen microRNA target prediction algorithms were used to identify microRNAs predicted to regulate 11 genes. The 3′-UTR of the 6 genes which topped the list of potential microRNA targets was sequenced in 30 black South Africans. In addition, genetic variants within these genes were investigated for interference with mRNA-microRNA interactions. Potential effects of observed variants were determined using *in silico* prediction tools.

**Results:** The 11 genes coding for DMEs, transporters and nuclear receptors were predicted to be targets of microRNAs with *CYP2B6, NR1I2 (PXR), CYP3A4*, and *CYP1A2*, interacting with the most microRNAs. The majority of identified genetic variants were predicted to interfere with microRNA regulation. For example, the variant, *rs1054190C* in *NR1I2* was predicted to result in the presence of a binding site for the microRNA miR-1250-5p, while the variant *rs1054191G* was predicted to result in the absence of a recognition site for miR-371b-3p, miR-4258 and miR-4707-3p. Fifteen of the seventeen, novel variants occurred within microRNA target sequences.

**Conclusion:** The 3′-UTR harbors variation that is likely to influence regulation of specific genes by microRNA. *In silico* prediction followed by functional validation could aid in decoding the contribution of variation in the 3′-UTR, to some unexplained heritability that affects drug response. Understanding the specific role of each microRNA may lead to identification of markers for targeted therapy and therefore improve personalized drug treatment.

## Introduction

Drug metabolizing enzymes (DMEs), drug transporters and nuclear receptors, play an important role in response outcomes to therapeutically prescribed drugs. For example, the analgesic drug codeine is dependent on CYP2D6 metabolism to the more active morphine metabolite (Armstrong and Cozza, 2003). However, CYP2D6 exhibits extensive genetic polymorphism resulting in individuals being broadly classified as poor metabolizers (PMs), extensive metabolizers (EMs), and ultrarapid metabolizers, phenotypes that vary across world populations. Another example is that of the disposition of efavirenz and nevirapine used in treatment of HIV/AIDS, both of which are metabolized by CYP2B6. The genetic variants *CYP2B6 c.516T*, and c.*983C* have been linked to high efavirenz or nevirapine plasma levels and the development of central nervous system side effects (Marzolini et al., 2001; Ciccacci et al., 2013; Swart et al., 2013; Vardhanabhuti et al., 2013). In general, many pharmacogenetics studies have concentrated on the effects of genetic variation in exons, introns, and promoters of pharmacogenetically relevant genes (Batt et al., 1994; Commandeur et al., 1995; Tukey and Strassburg, 2000; McCarver and Hines, 2002; Hayes et al., 2005; Petzinger and Geyer, 2006; Alnouti and Klaassen, 2008; Guengerich, 2008). However, it is now apparent that genetic variation in the 3′-UTR, which is a target for microRNA regulation, could also play a role in pharmacogenomics (Xu et al., 2005).

Thus, in order to get a complete picture of the pharmacogenetic determinants of drug response, genetic variation in the 3′-UTR region of DMEs, drug transporters and nuclear receptors, should also be evaluated as this is the region targeted by microRNAs. MicroRNAs are evolutionary conserved (Lagos-Quintana et al., 2001) and predicted to regulate more than 30% of protein coding genes in the human genome (Yu, 2009; Breving and Esquela-Kerscher, 2010; Shomron, 2010; Rodrigues et al., 2011) which include genes with pharmacogenomics relevance. MicroRNAs regulate genes post-transcriptionally by binding to the 3′-UTR of mRNA target sequences and facilitating degradation or translational repression (Baek et al., 2008; Mishra and Bertino, 2009; Marin and Vanicek, 2012). MicroRNAs have been implicated in regulation of the following pharmacogenomically relevant genes; *CYP1B1* (Tsuchiya et al., 2006), *CYP2E1* (Mohri et al., 2010), *CYP3A4* (Pan et al., 2009), and PXR (Takagi et al., 2008). Pan et al. (2009) showed that microRNA miR-27b regulates expression of *CYP3A4* directly, while miR-148a regulates *CYP3A4* indirectly through regulation of PXR (Takagi et al., 2008). *SULT1A1* has also been shown to be regulated directly by miR-631 in an allele-specific manner (Yu et al., 2010). Genes coding for *CYP1A2* and *CYP2B6*, are predicted to be regulated by microRNAs based solely on the length of their 3′-UTRs, but these predictions are yet to be confirmed through functional studies (Ingelman-Sundberg et al., 2007; Ramamoorthy and Skaar, 2011; Singh et al., 2011; Mishra, 2012).

Genetic variation that occurs either in the sequence coding for microRNAs or in the sequence targeted by microRNA on the mRNA, is referred to as miRSNPs. MiRSNPs potentially alter microRNA function or interfere with microRNA-mRNA interaction (Mishra et al., 2008; Liu et al., 2012). MiRSNPs located in the mRNA can result in the presence or absence of microRNA target sites and to date only a few pharmacogenomics studies have included genetic variation in the 3′-UTR of pharmacogenomically-relevant genes as possible contributors to interindividual variability observed in drug response. In order to evaluate the role of microRNA in drug response, prediction of likely microRNA target sequences should be followed by functional validation.

There are several algorithms to predict microRNA targets and these algorithms are designed based on a number of different criteria (Witkos et al., 2011). To increase the precision and power of microRNA target prediction as well as reduce false positives, the use of a combination of prediction algorithms is advised (Mishra and Bertino, 2009; Marin and Vanicek, 2012). Recently, a study by Ramamoorthy and Skaar (2011) used six algorithms to predict microRNAs that potentially target 12 major drug metabolizing CYPs. With more than 10 algorithms available, the challenge is which combinations or how many algorithms should be used to predict microRNA targets. The use of a combination of microRNA target prediction, using different features including; sequence complementarity, target site accessibility, binding energy, and conservation of target sites, in a complementary manner may improve sensitivity, specificity, and consistency of microRNA target prediction (Zhang and Verbeek, 2010). In this study, we set out to identify microRNAs predicted to regulate genes associated with drug metabolism and disposition using 15 *in silico* prediction algorithms that are available online to evaluate the spectrum of microRNA target sequences in the 3′-UTR of 11 genes. Variation in the 3′-UTR of six genes was evaluated by sequencing resulting in the identification of microRNA SNPs (miRSNPs) which were predicted to potentially affect microRNA binding.

## Methods

### Bioinformatics analysis to predict microRNA target sites in genes coding for drug metabolizing enzymes

Fifteen different web-based bioinformatics algorithms (DIANA-microT-CDS, DIANA-microT, MicroCosm, miR2Gene, miRanda-MirSVR, miRBRIDGE, miRSystem, miRTar, PACCMIT, PicTar, PITA, RegRNA, RNA22, TargetScan, and TargetSpy) that were freely-available online were used to predict microRNA targeting (John et al., 2004; Krek et al., 2005; Griffiths-Jones et al., 2006; Huang et al., 2006; Miranda et al., 2006; Kertesz et al., 2007; Sturm et al., 2010; Tsang et al., 2010; Hsu et al., 2011; Qiu et al., 2011; Lu et al., 2012; Vlachos et al., 2012) (Supplementary Table S1). These bioinformatics algorithms were selected to predict microRNA targeting based on a number of different features including; sequence complementarity, target site accessibility, binding energy, and conservation of target sites in order to increase precision and power.

The total number of unique microRNAs were calculated for each gene and the percentage overlap was estimated based on the number of microRNAs predicted by at least two algorithms. Analysis using these prediction algorithms was performed using the most recent version and respective default settings. Eight datasets containing microRNA expression profiles in liver tissue was obtained from mimiRNA (http://mimirna.centenary.org.au/mep/formulaire.html) (Ritchie et al., 2010) and compared to microRNAs predicted by the different algorithms and microRNAs with targeting predicted to be affected by miRSNPs, to determine overlap between predicted and functionally relevant microRNAs.

### Investigation of genetic variants within 3′-UTR and its potential effects on microRNA target sites

After prediction of likely microRNAs targeting genes coding for DMEs, the top six genes (with the longest 3′-UTR, most microRNA targets as well genes of interest in our research group) were chosen for sequencing of the 3′-UTR among 30 Bantu-speaking South Africans, and these genes included *CYP1A2, CYP2B6, CYP2D6, CYP3A4, NR1I2*, and *UGT2B7*. The assumption for selection of the six genes was based on the prediction that a gene with a longer 3′-UTR is more likely to be regulated by a larger number of microRNAs.

### Study participants

Participants were South African HIV/AIDS patients receiving efavirenz-based treatment for at least 6 months and recruited to participate in pharmacogenomics research. All subjects were of Bantu origin from Gauteng Province, South Africa. Participants gave information on their ethnicity, age, health status (including self-reported adherence to treatment or pill counts), dietary, and smoking habits. Ethics and study approval (HREC REF 103/2009) was given by the University of Cape Town, Faculty of Health Sciences Research Ethics Committee. Written informed consent was obtained from participants as part of a study focussing on pharmacogenetics of HIV therapy. Patients were mostly female (90%) and had an average age of 42 years. The research was performed in accordance with guidelines of the Helsinki Declaration of 2008.

### DNA isolation and sequencing

DNA was isolated from a 5 mL blood sample using the method adapted from Gustafson et al. (1987) or the GenEluteTM Blood Genomic DNA kit (Sigma-Aldrich, St Louis, Missouri, USA). Primers for each 3′-UTR were designed to flank the 3′-UTR and were specific to the gene of interest (designed by aligning the gene sequence with that of pseudogenes to select a region that is unique to the gene of interest). NCBI Primer-BLAST (Altschul et al., 1990) (http://www.ncbi.nlm.nih.gov/tools/primer-blast/), and IDT Oligo Analyzer from Integrated DNA Technologies (http://www.idtdna.com/analyzer/Applications/OligoAnalyzer/) were used for primer design while synthesis was done by Integrated DNA Technologies, Inc. (Coralville, Iowa, USA). Primers for each gene's 3′-UTR were as follows: *CYP1A2* (two sets), F1: 5'-CGACCTGACCCCCATCTAC-3′/R2: 5'-AAGAATGTAAGTTAGGCTGGATGTG-3′ and F2: 5'-CGCAGGTTCAAGCAATCC-3′/R1: 5'-AGGACTCAAGCACCAAGAGC-3′; *CYP2B6* (two sets), F1: 5'-GTGGTGCCATCTCTGTCCA-3′/R1: 5'-AGAGTTGGCATTGAGGTGAGAG-3′ and F2: 5'-GGCAAAATACCCCCAACATA-3′/R2: 5'-GCCTGTGATACCAGCTCCTC-3′; *CYP2D6*, F: 5'-GCCACCATGGTGTCTTTGCTTTCCTGG-3′/R: 5'-ACTGAGCCCTGGGAGGTAGGTAG-3′; *CYP3A4*, F: 5'-CACTGAAGGCGTGTCTCACTCACT-3′/R: 5'-CTTCTCTACCTTAATGTGAGGGCACCA-3′; *NR1I2*, F: 5'-CCAGGACATACACCCCTTTG-3′/R: 5'-TATTTCCACACCCCCACATT-3′; and *UGT2B7*, F: 5'-AGAGAGGAGTCTTGCCGATG-3′/R: 5'-GAGAATAAAGTCAACCAGATGT-3′, respectively.

PCR amplification was performed using the following conditions: initial denaturation at 94°C for 3 min, followed by 40 cycles of denaturation at 94°C for 30 s, annealing at 61°C (*CYP1A2* and *CYP2B6*), 60°C (*CYP3A4* and *NR1I2*), 58°C (*UGT2B7*), or 70°C (*CYP2D6*) for 30 s, primer extension at 72°C for 2 min and final extension at 72°C for 10 min. A “T100™ Thermal cycler” (Bio-Rad, Hercules, USA) was used and the PCR reaction contained the following reagents; 50–100 ng genomic DNA, 1X Green GoTaq Reaction Buffer (Promega Corporation, Madison, USA), 0.2 mM of each of the deoxynucleotide triphosphates (dNTPs) (Bioline, London, UK), 1.5 mM MgCl_2_ (except for *CYP1A2* fragment 1 where 2 mM MgCl_2_ was used), 40 pmol of the forward and reverse primers (Integrated DNA Technologies, Inc., Coralville, USA) and 2U of GoTaq DNA Polymerase (Promega Corporation, Madison, USA).

PCR product was cleaned using FastAP (Fermentas Life Sciences, Burlington, Canada) and *ExoI* (Fermentas Life Sciences, Burlington, Canada). The reaction conditions were as reported previously (Swart et al., 2013). The cleaned PCR product was sequenced on a “GeneAmp® PCR System 9700 version 3.08 (Applied Biosystems, Carlsbad, CA, USA) and capillary electrophoresis was performed on an ABI3130xl Genetic Analyzer (Applied Biosystems, Carlsbad, CA, USA). Analysis of the sequences was performed using DNAstar Lasergene Sequence Alignment Editor V.10 (DNASTAR, Inc., Madison, WI, USA).

### Bioinformatics prediction of potential effects of SNPs located in the 3′-UTR

The PolymiRTS database 3.0 (Bao et al., 2007; Ziebarth et al., 2012; Bhattacharya et al., 2014) (http://compbio.uthsc.edu/miRSNP/) was used to predict if SNPs located in the 3′-UTR of genes would affect mRNA-microRNA interaction. A search in the database was performed using the GeneID obtained from the NCBI database (http://www.ncbi.nlm.nih.gov/) and default settings. Pre-computed context+ scores were included in a recent update of TargetScan as a measure of predicted efficacy of microRNA targeting and down regulation of the target mRNA (log2 fold change in mRNA abundance after microRNA transfection assays) (Grimson et al., 2007) by summing up contributions made by individual sites using information on the site-type contribution, 3′-pairing contribution, local AU contribution, position contribution, target-site abundance contribution and seed-pairing stability contribution. The PolymiRTS database use the context+ scores from TargetScan to calculate the difference in context+ scores between the reference and derived alleles for each SNP. Differences in context+ scores caused by a SNP in the microRNA target site have been included in this study and a more negative difference in context+ scores indicates an increased likelihood that the target sites are either absent or present due to the variant allele, aiding in prioritizing miRSNPs with potential effect instead of showing miRSNPs that significantly alter microRNA binding (Bhattacharya et al., 2014).

To assess the potential effect of novel SNPs, the mrSNP software was used by selecting assembly hg19 of the human genome, using default cut-offs and inserting the chromosome position and two alleles. The prediction method applied is adapted from Diana-microT (Maragkakis et al., 2009; Deveci et al., 2014) (http://mrsnp.osu.edu/). Prediction is based on the ratio of binding energy and maximum binding energy of a microRNA (maximum MFE in Supplementary Table S3). The identified SNPs need to be further characterized in terms of their differential tissue expression as well as strength in achieving the predicted effects.

### Comparison of 3′-UTR genetic variation in different populations

Minor allele frequencies (MAF) for SNPs in the 3′-UTR of the 6 genes sequenced among the 30 black South Africans, were compared to those of other world population groups, obtained from the dbSNP database (http://www.ncbi.nlm.nih.gov/SNP/), the HapMap project (http://hapmap.ncbi.nlm.nih.gov/), and the 1000 genomes project (http://www.1000genomes.org/). Statistical analyses were performed using the Graphpad Prism statistical program (Version 5, GraphPad Software Inc., San Diego, CA). Pearson's χ^2^-test and Fisher's exact test were used to examine differences between the population groups with regards to distribution of minor alleles. For all statistical tests significance was defined as *P* < 0.05. The novel SNPs identified in the 3′-UTR of the six genes could not be compared to other world population groups as they have not been reported before.

## Results

### MicroRNAs predicted to target pharmacogenomically-relevant genes

*CYP1A2, CYP2B6, CYP2D6, CYP3A4, CYP3A5, GSTP1, UGT1A1, UGT2B7, SULT1A1, NR1I2*, and *NR1I3* were all predicted to be targets of microRNAs. The genes with the highest number of hits for microRNA target sites were *CYP2B6, NR1I2 (PXR), CYP1A2*, and *CYP3A4*, respectively (Table 1). *CYP2B6, CYP1A2*, and *CYP3A4* were also predicted to be targets of many microRNAs by Ramamoorthy and Skaar (2011). Extensive variability in the number of microRNAs predicted by each algorithm was observed as expected. For example, the program miRTar estimated only four microRNA targets in *CYP1A2*, whereas the program PITA, estimated 78 microRNA targets in the same gene. The algorithm TargetScan predicted the highest number of microRNA target sites while the algorithms, PICTAR, and miRTar predicted the least number of target sites. The overlap in the number of microRNAs predicted by 15 algorithms ranged from 14 to 30% and the four genes predicted to be targets of the highest number of microRNAs (*CYP1A2, CYP2B6, CYP3A4*, and *NR1I2*) had at least 93% of their microRNAs predicted by seven algorithms (miR2Gene—DIANA-microT v.3, miRanda-MirSVR, miRSystem, PACCMIT, PITA, TargetScan, and TargetSpy). The specific microRNAs predicted to target *CYP1A2, CYP2B6, CYP3A4*, and *NR1I2* by most algorithms are miR-542-3p (9 algorithms), miR-542-3p (10 algorithms), miR-548c-5p (11 algorithms), and miR-133a (9 algorithms), respectively (Table 2). The microRNAs predicted to target the most genes were miR-216a, miR-548a, miR-508-5p, miR-510, miR-671-5p, miR-142-3p, miR-608, miR-548b-5p, miR-548c-5p, miR-548d-5p, miR-548i, miR-548j, miR-514, miR-548a-3p, miR-548a-5p, miR-548h, miR-152, miR-148b, miR-214-3p, miR-520h, miR-520g, miR-330-5p, and miR-326. Comparison between the number of microRNAs predicted by the 15 algorithms and microRNAs expressed in liver tissue, showed an overlap of about 16% (240 microRNAs both expressed and predicted/1537 predicted microRNAs). Additionally, the length of the 3′-UTR correlated significantly (*r*^2^ = 0.764, *P*-value = 0.0062) with the number of microRNAs predicted to target the specific gene. The correlation of number of microRNAs predicted to target and the length of the 3′-UTR is similar to previous reports (Ramamoorthy and Skaar, 2011).

**Table 1:** ***In silico* prediction of microRNA targeting pharmacogenomically-relevant genes**.

	**CYP1A2**	**CYP2B6**	**CYP2D6**	**CYP3A4**	**CYP3A5**	**GSTP1**	**UGT1A1**	**UGT2B7**	**SULT1A1**	**NR1I2**	**NR1I3**
	**NC_000015.8 (NM_000761)**	**NC_000019.8 (NM_000767)**	**NC_000022.9 (NM_000106)**	**NC_000007.12 (NM_017460)**	**NC_000007.12 (NM_000777)**	**NC_000011.9 (NM_000852)**	**NC_000002.11 (NM_000463)**	**NC_000004.11 (NM_001074)**	**NC_000016.9 (NM_001055)**	**NC_000003.11 (NM_022002)**	**NC_000001.10 (NM_005122)**
3′-UTR length (bp)	1512	1569	75	1152	111	76	740	251	226	1273	131
Number of reported 3′-UTR SNPs	27	99	0	45	3	4	22	14	4	30	5
**Bioinformatic programs used**	**Number of microRNAs predicted to target the 3**'**-UTR of each gene**										
DIANA-microT-CDS	31	43	11	59	2	3	36	13	4	41	21
DIANA-microT v.4	3	28	52	12	0	4	0	0	0	30	48
MicroCosm	7	5	33	34	12	14	42	14	29	16	70
miR2Gene (DIANA-microT v.3)	84	122	72	82	3	6	N/A	13	12	107	50
miR2Gene (microcosm)	7	5	5	8	11	0	0	14	29	16	69
miRanda-MirSVR	92	108	4	101	101	4	86	38	20	113	19
miRBRIDGE	0	4	0	0	0	0	6	0	0	24	1
miRSystem	79	143	71	97	36	6	37	20	17	126	65
miRTar	4	5	1	7	2	3	4	1	1	3	3
PACCMIT (accessibility)	70	74	62	101	2	3	0	10	11	104	44
PACCMIT (accessibility and conservation)	5	0	14	0	0	0	0	0	0	3	13
PICTAR	0	0	0	0	0	0	3	0	0	0	0
PITA	78	82	4	68	2	1	50	20	9	77	8
RegRNA	22	19	7	15	9	10	12	10	5	14	6
RNA22	0	34	1	22	33	0	11	8	6	34	0
TargetScan (conserved)	0	9	0	13	1	0	4	0	0	9	0
TargetScan (non-conserved)	240	312	17	235	24	11	154	51	35	286	10
TargetSpy	53	42	1	35	1	0	21	4	5	22	2
Total number of microRNAs	775	1035	355	889	239	65	466	216	183	1025	429
Total number of unique microRNAs predicted by 16 programs	436	515	196	429	176	37	256	112	93	475	188
Overlap (%)	163 (21%)	223 (21.5%)	97 (27.3%)	192 (21.6%)	49 (20.5%)	9 (13.8%)	108 (23.2%)	53 (24.5%)	55 (30.1%)	231 (22.5%)	116 (27%)
Total number of unique microRNAs predicted by 16 programs	420 (96.3%)	504 (97.9%)	156 (79.6%)	403 (93.9%)	157 (89.2%)	16 (43.2%)	229 (89.5%)	94 (83.9%)	68 (73.1%)	460 (96.8%)	127 (67.6%)

Overlap (%) is the number of the same microRNAs picked by at least two algorithms, the selected seven algorithms are miR2Gene—DIANA-microT v.3, miRanda-MirSVR, miRSystem, PACCMIT, PITA, TargetScan, and TargetSpy.

**Table 2:** **MicroRNAs predicted to target the 3′-UTR of *CYP1A2, CYP2B6, CYP3A4*, and *NR1I2* by seven or more algorithms**.

**Ranking**	**CYP1A2**	**Position of microRNA target site**	**CYP2B6**	**Position of microRNA target site**	**CYP3A4**	**Position of microRNA target site**	**NR1I2**	**Position of microRNA target site**
	**NC_000015.8**	**NM_000761**	**NC_000019.8**	**NM_000767**	**NC_000007.12**	**NM_017460**	**NC_000003.11**	**NM_022002**
1	miR-542-3p^#^	394–400	miR-542-3p^#^	677–683, 912–918	miR-548c-5p	67–73	miR-133a^#^	351–357
2	miR-320a	95–102, 317–324	miR-485-5p	332–338, 759–765	miR-548d-5p	67–73	miR-133b^#^	351–357
3	miR-548c-3p	525–531, 903–909, 1204–1210	miR-661	256–262, 1067–1073	miR-548b-5p	67–73	miR-760	42–48
4	miR-128	925–931	miR-940	87–93, 325–331, 752–758	miR-608	261–267	miR-142-5p^#^	1169–1176
5	miR-143^#^	257–263, 1092–1099	miR-28-3p^#^	632–638	miR-455-3p	112–118	miR-34c-5p	84–91
6	miR-320b	95–102, 317–324	miR-590-3p^#^	186–192, 535–541, 561–568, 583–589	miR-508-5p	403–410	miR-449a	84–91
7	miR-485-5p	476–482, 845–851, 978–984, 1140–1147, 1146–1152	miR-656^#^	192–199, 1297–1303	miR-548a-5p	67–73	miR-532-5p	1178–1184
8	miR-650^#^	1115–1121, 1413–1419	miR-1257	549–555, 605–612	miR-206^#^	1087–1093	miR-580	1084–1091
9	miR-940	604–610, 838–844, 1411–1417, 1420–1426	miR-128	412–418,	miR-27a^#^	607–613	miR-769-3p^#^	322–328
10			miR-134^#^	1372–1379	miR-369-3p^#^	455–461, 998–1004	miR-18b^#^	1057–1064
11			miR-1827	88–94, 326–333, 753–760, 1414–1420	miR-499-5p	937–944	miR-221^#^	1212–1218
12			miR-216a	175–182	miR-548i	67–73	miR-34a^#^	84–91
13			miR-331-5p^#^	900–906	miR-548j	67–73	miR-514	334–340, 1230–1236
14			miR-489	841–847	miR-559	67–73	miR-522^#^	1046–1053
15			miR-504^#^	31–38	miR-569	278–284, 583–589	miR-607^#^	637–643
16			miR-548c-3p	390–396, 817–823, 1027–1033	miR-765^#^	284–290, 328–334	miR-640^#^	762–769
17			miR-548d-3p	963–969	miR-944	321–327		
18			miR-767-5p	1083–1090				
19			miR-920	1144–1151				

# MicroRNAs also confirmed to be functionally expressed in liver tissue.

### Genetic variants identified by sequencing of the 3′-UTR of genes of interest

Based on the total microRNAs predicted to target the mRNA, the top four genes *CYP1A2, CYP2B6, CYP3A4*, and *NR1I2* were selected for further analysis. *CYP2D6* and *UGT2B7* were included as they form part of our ongoing pharmacogenomics studies. In total six genes had their 3′-UTR sequenced among 30 individuals. A total of 52 genetic variants were identified, including 17 novel and 35 previously reported SNPs. Novel SNPs identified, despite comprehensive re-sequencing of various population groups, are potentially rare and population specific or this may be an indication of the exclusion of populations from this region in the re-sequencing projects. *CYP2B6* had the most genetic variants in the 3′-UTR accounting for 26 of the 52. No SNPs were identified within the 75bp 3′-UTR of *CYP2D6* (Table 3). Forty of the 52 genetic variants were predicted to potentially affect regulation of 212 microRNAs by creating or abolishing microRNA target sites depending on which of the variants is the ancestral variant (Supplementary Table S2). Based on predictions by mrSNP, 15 of the 17 novel SNPs were located within microRNA target sites and potentially result in the creation of microRNA target sites (Supplementary Table S3). Comparison between the number of microRNAs potentially affected by miRSNPs and microRNAs expressed in liver tissue, showed an overlap of about 3% (10 microRNAs both expressed and predicted to potentially be affected by miRSNPs/363 expressed microRNAs).

**Table 3:** **Genetic variation identified in the 3′-UTR of *CYP1A2*, *CYP2B6*, *CYP2D6*, *CYP3A4*, *NR1I2*, and *UGT2B7* after sequencing of 30 black South Africans**.

**Gene**	**NCBI reference sequence**	**Variation**
CYP1A2	NC_000015.10	Known: rs11636419, rs56141902, rs45564134, rs34002060, rs45599945, rs17861162
		Novel: g.74755658G>A, g.74756006G>A, g.74756039T>A, g.74756176G>A, g.74756438T>C
CYP2B6	NC_000019.10	Known: rs28399502, rs3181842, rs138892132, rs7246465, rs148726498, rs4803420, rs35742808,, rs28969420, rs35462975, rs28969421, rs1038376, rs3211398, rs707265, rs70950385, rs1042389
		Novel: g.41016968T>A, g.41017103C>T, g.41017242T>C, g.41017290A>C, g.41017450A>T, g.41017478C>T, g.41017486C>T, g.41017525A>T, g.41017749C>A, g.41017763C>A, g.41017847A>G
CYP2D6	NC_000022.11	None
CYP3A4	NC_000007.14	Known: rs28988604, rs33972239
		Novel: g.99784127T>C
NR1I2	NC_000003.12	Known: rs3732358, rs3732359, rs10511395, rs3732360, rs1054190, rs6438550, rs1054191, rs3814057, rs3814058
UGT2B7	NC_000004.12	Known: rs6851533, rs6600893, rs150516790

rs# of SNPs are according to annotation in dbSNP.

### Comparison of minor allele frequencies between different populations

Of the 52 variants identified in sequencing the 3′-UTR of selected genes among South African black participants, 18 SNPs which also had information for other population groups (i.e., through the HapMap and 1000 genomes projects) were compared to other African groups, African-Americans, Asian, and European population groups (Table 4). Statistically significant differences were observed when the South African group was compared to Asian, Caucasian and other Africa groups. For example, *NR1I2 rs1054190T* allele occurring at a frequency of 0.18 in the South African group, was absent among Asians, significantly lower among another African group the Yoruba (0.02), yet comparable to that reported among Caucasians (0.15). For example, the variant, *rs1054190C* in *NR1I2* was predicted to result in the presence of a binding site for the microRNA miR-1250-5p, while the variant *rs1054191G* was predicted to result in the absence of a recognition site for miR-371b-3p, miR-4258, and miR-4707-3p. A second SNP in *NR1I2, rs3732359A* allele showed no significant differences when the South African group (0.56) was compared to Asians (0.47), but showed statistically significant differences when compared to the Caucasians (0.84) and Yoruba (0.24). As expected, differences in allele frequencies of miRSNPs were observed when comparing the South African Bantu population group to both Caucasian and Asian populations as highlighted by the *CYP2B6 rs28969420T* allele (20, 4, 3%, respectively).

**Table 4:** **Comparison of minor allele frequencies for selected genetic variants in the 3′-UTR of pharmacogenomically-relevant genes among different world populations**.

**dbSNP rs^#^**	**N**	**rs11636419**	**rs45564134**	**rs17861162**	**rs1038376**	**rs1042389**	**rs707265**	**rs3181842**	**rs7246465**	**rs28969420**	**rs10511395**	**rs1054190**	**rs1054191**	**rs3732359**	**rs3732360**	**rs3814057**	**rs3814058**	**rs6600893**	**rs6851533**
**Allele**		**G**	**–**	**G**	**T**	**C**	**A**	**C**	**T**	**T**	**A**	**T**	**A**	**A**	**T**	**C**	**C**	**C**	**C**
South African	30	0.24	0.12	0.15	0.34	0.15	0.23	0.25	0.29	0.20	0.14	0.18	0.26	0.56	0.52	0.25	0.27	0.28	0.36
AFR	691	0.14	0.16	0.16	0.31	0.28	0.18	0.24	0.21	0.23	0.16	0.04^*^	0.16	0.34^*^	0.33^*^	0.47^*^	0.47^*^	0.19	0.19^*^
ASW	66	0.17	0.15	0.19	0.31	0.2	0.21	0.28	0.26	0.15	0.17	0.07	0.17	0.44	0.43	0.42	0.42	0.24	0.24
LWK	116	0.13	0.16	0.16	0.3	0.36^*^	0.16	0.24	0.18	0.30	0.22	0.05^*^	0.21	0.37	0.35	0.47^*^	0.47^*^	0.17	0.17^*^
YRI	116	0.14	0.16	0.16	0.33	0.24	0.18	0.24	0.21	0.21	0.1	0.02^*^	0.10^*^	0.24^*^	0.24^*^	0.51^*^	0.51^*^	0.15	0.15^*^
MKK^#^	180	N/A	N/A	N/A	N/A	0.24	0.21	0.02^*^	N/A	N/A	0.1	0.05^*^	0.09^*^	0.45	0.45	0.38	N/A	0.29	0.29
AMR	355	0.08^*^	0.01^*^	0.08	0.43	0.16	0.22	0.22	0.23	0.07^*^	0.15	0.11	0.15	0.76^*^	0.73^*^	0.2	0.2	0.35	0.35
CLM	95	0.10	0.01^*^	0.10	0.48	0.14	0.18	0.18	0.20	0.11	0.18	0.13	0.18	0.76^*^	0.73^*^	0.18	0.18	0.43	0.43
MXL	69	0.09^*^	0^*^	0.09	0.39	0.15	0.23	0.23	0.23	0.04^*^	0.13	0.08	0.13	0.78^*^	0.74^*^	0.2	0.2	0.27	0.27
PUR	105	0.04^*^	0.01^*^	0.04^*^	0.42	0.19	0.25	0.25	0.25	0.07^*^	0.15	0.1	0.14	0.75^*^	0.72^*^	0.21	0.21	0.36	0.37
GIH^#^	100	N/A	N/A	N/A	N/A	0.22	0.19	0^*^	N/A	N/A	0.1	0.06	0.07^*^	0.45	0.45	0.28	N/A	0.48^*^	0.48
ASN	523	0.22	0^*^	0.22	0.14^*^	0.28	0.35	0.35	0.33	0.03^*^	0^*^	0^*^	0^*^	0.47	0.46	0.48^*^	0.48^*^	0.28	0.28
CHB	106	0.27	0.01^*^	0.27	0.12^*^	0.31	0.34	0.33	0.32	0.04^*^	0^*^	0^*^	0^*^	0.49	0.49	0.46	0.46	0.31	0.31
CHS	112	0.22	0^*^	0.22	0.16^*^	0.26	0.37	0.37	0.33	0.02^*^	0.01^*^	0^*^	0.01^*^	0.39	0.38	0.55^*^	0.55^*^	0.25	0.25
JPT	105	0.17	0.01^*^	0.17	0.14^*^	0.28	0.34	0.37	0.36	0.02^*^	0^*^	0^*^	0^*^	0.52	0.52	0.44	0.44	0.29	0.29
CHD^#^	100	N/A	N/A	N/A	N/A	0.32	0.36	0^*^	N/A	N/A	0.01^*^	0^*^	0^*^	0.44	0.44	0.48^*^	N/A	0.32	0.32
EUR	514	0.08^*^	0^*^	0.07	0.26	0.21	0.39	0.40	0.38	0.06^*^	0.16	0.13	0.16	0.79^*^	0.76^*^	0.17	0.17	0.49^*^	0.49
CEU	103	0.04^*^	0^*^	0.04^*^	0.31	0.19	0.39	0.42	0.38	0.04^*^	0.18	0.15	0.16	0.84^*^	0.81^*^	0.12^*^	0.12^*^	0.48^*^	0.48
FIN	100	0.11	0^*^	0.10	0.17	0.19	0.43	0.43	0.43	0.06^*^	0.06	0.05^*^	0.06^*^	0.72	0.7	0.21	0.21	0.45	0.45
GBR	94	0.06^*^	0^*^	0.06	0.25	0.26	0.39	0.41	0.39	0.05^*^	0.19	0.16	0.19	0.78^*^	0.75^*^	0.17	0.17	0.48^*^	0.48
IBS	107	0.04^*^	0^*^	0.04^*^	0.25	0.36^*^	0.29	0.29	0.29	0.07^*^	0.11	0.07	0.11^*^	0.86^*^	0.82^*^	0.14	0.14	0.46	0.46
TSI	110	0.09^*^	0^*^	0.09	0.32	0.18	0.36	0.36	0.36	0.08	0.21	0.18	0.21	0.8^*^	0.76^*^	0.18	0.18	0.48^*^	0.48

* Indicate significant differences in allele frequency compared to the South Africans,

# Allele frequencies were obtained from the HapMap project, while allele frequencies for other populations were obtained from the 1000 genomes project, AFR, African; ASW, Americans of African ancestry in SW USA; LWK, Luhya in Webuye (Kenya); YRI, Yoruba in Ibadan (Nigeria); MKK, Maasai in Kinyawa (Kenya); AMR, Ad mixed American; CLM, Colombians from Medellin (Colombia); MXL, Mexican ancestry from Los Angeles USA; PUR, Puerto Ricans from Puerto Rico; GIH, Gujarati Indian from Houston Texas; ASN, East Asian; CHB, Han Chinese in Bejing (China); CHS, Southern Han Chinese; JPT, Japanese in Tokyo (Japan); CHD, Chinese in Metropolitan Denver (Colorado); EUR, European; CEU, Utah residents with Northern and Western European ancestry; FIN, Finnish in Finland; GBR, British in England and Scotland; IBS, Iberian population in Spain; TSI, Toscani in Italy; N/A, not available.

## Discussion

### MicroRNAs predicted to target genes coding for drug metabolizing enzymes

Pharmacogenomics studies have focussed largely on the effects of genetic variation in genes coding for DMEs (Ingelman-Sundberg et al., 2007) and very few have investigated the role of variation at the level of microRNA target sites. MicroRNAs affect global gene expression, thus, including miRSNPs in pharmacogenomic tests, could potentially explain some of the genetics-associated differences in drug response (Mishra and Bertino, 2009). Computational prediction analysis carried out in this study has highlighted microRNAs that regulate some of the genes with pharmacogenomics relevance, further confirming observations by Ramamoorthy and Skaar (2011) with respect to *CYP1A2, CYP2B6*, and *CYP3A4*.

At least 10% more microRNAs were predicted to target DMEs, for example the genes *CYP1A2, CYP2B6, CYP2D6, CYP3A4*, and *CYP3A5* were reported to be targeted by 436, 515, 196, 429, and 176 potential microRNAs using the 15 prediction algorithms, compared to 386, 416, 40, 333, and 32 using six algorithms as reported by Ramamoorthy and Skaar (2011). In addition, the number of microRNAs predicted using TargetScan v.6.2 in the current study were more than double those reported by Ramamoorthy, using an earlier version (Ramamoorthy and Skaar, 2011). The higher number of microRNAs predicted, is potentially a result of using newer versions of the algorithms compared to those used by Ramamoorthy and Skaar (2011). The use of more algorithms was justified by the low percentage (14–30%) overlap between microRNAs predicted by two or more algorithms. However, the question still remains as to what is the appropriate minimum number or combination of algorithms to pick most microRNAs. MicroRNA prediction using 7 of the 15 algorithms (miR2Gene—DIANA-microT v.3, miRanda-MirSVR, miRSystem, PACCMIT, PITA, TargetScan, and TargetSpy), showed on average 83% (range 43–98%) of all unique microRNAs, pointing toward the major contribution of these seven algorithms (especially TargetScan) compared to the remaining eight algorithms. The appropriate combination of algorithms will only be apparent after confirmatory functional validation of the predicted binding targets.

A few microRNAs have been experimentally shown to regulate expression of DMEs. For example, Pan et al. (2009) reported that *CYP3A4* is regulated by miR-27b directly, and by miR-148a indirectly through the regulation of PXR (Takagi et al., 2008). This observation is confirmed by our prediction analysis as five algorithms predicted miR-27b to target *CYP3A4*, while four algorithms predicted miR-148a to target *NR1I2* (coding for PXR). Two microRNAs namely; miR-590 and miR-27a have been associated with either targeting *CYP2B6* (miR-590) or *CYP3A4* (miR-27a) (Ramamoorthy et al., 2013). The finding of *CYP2B6, CYP3A4*, and *UGT1A1* being targets of miR-590, miR-27a, miR-491-3p, respectively (Dluzen et al., 2014), is supported by our prediction analysis. However, our observations on *SULT1A1* mRNA are contradictory to those of Yu et al. (2010), who reported targeting by miR-631, yet none of the 15 computational algorithms picked it. MiR-26b-5p is reportedly associated with *CYP2D6* expression (Gennarino et al., 2009) and miR-335-5p with *CYP3A5*, but none of the two microRNAs was picked by the 15 algorithms used in the current prediction analysis. It appears that the prediction algorithms might have inherent weaknesses, for example, *GSTP1* has been experimentally validated to be affected by miR-513a-3p, miR-133a-3p, miR-26b-5p, and miR-92a-3p, but only miR-133a-3p was picked by the use of 15 algorithms. It is important to note that computational prediction algorithms are thought to predict microRNA-mRNA targeting with a fraction of false positives estimated at 31% and it is, thus, of importance to prioritize microRNAs for further studies (Lewis et al., 2003). The overlap between microRNAs predicted by the 15 algorithms and microRNAs potentially affected by miRSNPs with microRNAs expressed in liver tissue is very low. Prediction algorithms use different criteria to rank microRNAs that potentially target mRNA of a gene of interest, and are likely to be more lenient in the prediction as it is mostly based on sequence complementarity, target site accessibility, binding energy and conservation of target sites. Also on the other hand, expression is dependent on many other factors including export and tissues involved. Further comprehensive microRNA expression profiling in liver as well as extrahepatic tissues would contribute toward identifying microRNAs that are potentially involved in regulation of DMEs. There is also a strong need for experimental validation of predicted microRNA targets.

### Genetic variants identified after sequencing the 3′-UTR and comparison of minor allele frequencies in the south african group to other world population groups

A total of 52 SNPs were identified within the 3′-UTR of *CYP1A2, CYP2B6, CYP3A4, NR1I2*, and *UGT2B7* including 17 novel SNPs, which is an indication of the extensive degree of genetic diversity characteristic to African and specifically South African populations (Matimba et al., 2009; Warnich et al., 2011; Dandara et al., 2014). SNPs (miRSNPs) identified in the South African group and already available in the PolymiRTS database, were predicted to alter the microRNA targeting of more than 200 microRNAs (Supplementary Table S2). As expected, differences in allele frequencies of miRSNPs were observed when comparing the South African Bantu population group to both Caucasian and Asian populations as highlighted by the *CYP2B6 rs28969420T* allele (20% compared to 4 and 3%, respectively) and the *NR1I2 rs3732359A*-allele (59% compared to 84 and 47%, respectively). Differences in the frequency of miRSNPs among population groups as highlighted by the *NR1I2 rs37372359A* allele, which results in the presence of a target site for several microRNAs including miR-362-5p, miR-500b-5p, and miR-501-5p, could translate into population differences in drug disposition and response. These qualitative and quantitative differences in allele frequencies of SNPs further support previous studies arguing against directly inferring effects of therapeutic drugs from one population to another (Swart et al., 2012a,b,c; Dandara et al., 2014). The 3′-UTR variation identified in the current study contributes to further genetic characterizing of South Africans. However, the observed variation should be functionally validated in order to deduce its contribution to the observed variability in drug response.

### Therapeutic potential of microRNAs

Response to therapeutic treatment is a complex phenotype determined by genetic variation in DMEs, nuclear receptors, drug transporters, miRSNPs, and pharmacoepigenetics (Zhang et al., 2010). MicroRNAs have the potential to be used as therapeutic targets through their ability to regulate expression of pharmacogenomically relevant genes (Singh et al., 2011; Van Rooij et al., 2012). Creating a microRNA recognition site leads to down regulation of mRNA expression of specific genes, while disruption of a site causes loss of down regulation of mRNA expression (Mishra and Bertino, 2009).Caution should however be exercised when developing microRNA-based therapeutics because of unpredictable off-target effects due to microRNAs having broad mRNA targets (Singh et al., 2011). A study by Mishra et al. (2007) demonstrated the effect of a SNP near the miR-24 binding site in the 3′-UTR of the dihydrofolate reductase (*DHFR*) gene, that interferes with miR-24 binding and resulting in DHFR overexpression and methotrexate resistance. Resistance to the cancer therapeutics cisplatin and 5-fluorouracil has been linked to induced miR-148a expression and targeting of the gene KIT (Hummel et al., 2011). MiR-148a has been shown by Takagi et al. (2008) to regulate PXR and affect downstream expression of *CYP3A4*, resulting in reduced drug metabolism. Presence of the *NR1I2 rs1054190T* allele results in the absence of a miR-148a target site, potentially leading to increased *CYP3A4* expression. It is important that future studies investigate the functional significance of microRNAs and miRSNPs. The recently developed Clustered Regularly Interspaced Short Palindromic Repeats (CRISPR) technology could be used to create knockout cell lines for specific microRNAs to validate their role in targeting of genes with pharmacogenomics relevance (Dandara et al., 2014).

## Conclusion

The integration of microRNA variation and epigenetics into pharmacogenomics studies may provide additional insights into the mechanisms of drug response and advance individualized therapy (Welsh et al., 2009; Zhang and Dolan, 2010). Use of *in silico* methods should be complimented by functional validation to confirm the predicted effects. It is therefore possible that some of the observed non-reproducibility of pharmacogenomics studies could partially be due to the unexplained variation in the 3′-UTR, thus, functional validation of microRNA targets should be another area of research to advance pharmacogenomics.

## Author contributions

Marelize Swart carried out the *in silico* prediction and sequencing analysis and drafted the manuscript. Collet Dandara conceived of the study, designed, coordinated the study, and also assisted with statistical data analysis, helped to draft the manuscript and approved the final version. Both authors read and approved the final manuscript.

### Conflict of interest statement

The authors declare that the research was conducted in the absence of any commercial or financial relationships that could be construed as a potential conflict of interest.
