# Supplementary material for: Genetic variation in the 3′-UTR of CYP1A2, CYP2B6, CYP2D6, CYP3A4, NR1I2, and UGT2B7: potential effects on regulation by microRNA and pharmacogenomics relevance
Source: Front Genet. 2014 Jun 4;5:167. doi: 10.3389/fgene.2014.00167 (PMC4044583; doi:10.3389/fgene.2014.00167)
Supplement: Supplementary file 1 [file DataSheet1.ZIP › 80478_Dandara_Data_Sheet_1.DOCX]

**Supplementary Table S1: Prediction algorithms and program settings used in predicting microRNAs targeting genes coding for drug metabolising enzymes**

| **Algorithm** | **Reference** | **Web address/link** | **Predictive programs settings** |
| --- | --- | --- | --- |
| DIANA-microT-CDS v.5 | Vlachos et al., 2012 | http://diana.imis.athena-innovation.gr/DianaTools/index.php?r=microT_CDS/index | A threshold of 0.6 was used for the target prediction score |
| DIANA-microT v.4 | Vlachos et al., 2012 | http://diana.cslab.ece.ntua.gr/DianaTools/index.php?r=microtv4/index | A threshold of 0.3 was used for the target prediction score |
| microcosm Targets, (formerly referred to as miRBase Targets) v.5 | Griffiths-Jones et al., 2006 | http://www.ebi.ac.uk/enright-srv/microcosm/htdocs/targets/v5/ | *Homo sapiens* and mRNA reference IDs obtained from the website of the National Center for Biotechnology Information |
| miR2Gene microcosm and DIANA-microT v.3 | Qiu et al., 2011 | http://202.38.126.151/hmdd/tools/mir2gene.html and http://diana.cslab.ece.ntua.gr/ | *Homo sapiens* and mRNA reference IDs obtained from the website of the National Center for Biotechnology Information |
| miRanda | John et al., 2004 | http://microrna.org/microrna/get-GeneForm.do | *Homo sapiens* and mRNA reference IDs obtained from the website of the National Center for Biotechnology Information |
| miRBRIDGE | Tsang et al., 2010 | http://mirbridge.org/ | *Homo sapiens* and mRNA reference IDs obtained from the website of the National Center for Biotechnology Information |
| miRSystem v.20130328 | Lu et al., 2012 | http://mirsystem.cgm.ntu.edu.tw/ | *Homo sapiens* and mRNA reference IDs obtained from the website of the National Center for Biotechnology Information |
| miRTar | Hsu et al., 2011 | http://mirtar.mbc.nctu.edu.tw/human/ | An alignment score ≥ 170 and a Minimum Free Energy (MFE) score of ≤ -14 Kcal/mol was used |
| PACCMIT | Marin and Vanicek, 2012 | http://lcpt.epfl.ch/MicroRNA_target_predictions | Both accessibility and accessibility + conservation filters were used |
| PicTar | Krek et al., 2005 | http://pictar.mdc-berlin.de/ | *Homo sapiens* and mRNA reference IDs obtained from the website of the National Center for Biotechnology Information |
| PITA | Kertesz et al., 2007 | http://genie.weizmann.ac.il/pubs/mir07/mir07_prediction.html | A minimum seed size of 8 nucleotides were used, a single G:U base pairing and a single mismatch was allowed with no flanking sequence |
| RegRNA | Huang et al., 2006 | http://regrna.mbc.nctu.edu.tw/html/about.html | An alignment score ≥ 165 and a Minimum Free Energy (MFE) score of ≤ -7 Kcal/mol was used |
| RNA22 | Miranda et al., 2006 | http://cbcsrv.watson.ibm.com/rna22.html | A maximum number of un-paired bases of 0 in seed/nucleus of 6 nucleotides, minimum number of paired-up bases in heteroduplex of 14 and maximum folding energy for heteroduplex of -25 Kcal/mol was used |
| TargetScan v.6.2 | Lewis et al., 2003 | http://www.targetscan.org/ | Both conserved and non-conserved microRNAs were included in analysis |
| TargetSpy v.1 | Sturm et al., 2010 | http://www.targetspy.org/ | *Homo sapiens* and mRNA reference IDs obtained from the website of the National Center for Biotechnology Information |
