# Supplementary material for: Genetic variation in the 3′-UTR of CYP1A2, CYP2B6, CYP2D6, CYP3A4, NR1I2, and UGT2B7: potential effects on regulation by microRNA and pharmacogenomics relevance
Source: Front Genet. 2014 Jun 4;5:167. doi: 10.3389/fgene.2014.00167 (PMC4044583; doi:10.3389/fgene.2014.00167)
Supplement: Supplementary file 1 [file DataSheet1.ZIP › 80478_Dandara_Data_Sheet_2.DOCX]

**Supplementary Table S2: Potential effect of genetic variation identified in the 3'-UTR on microRNA targeting**

| **dbSNP rs#** | **Allele** | **MicroRNA ID (hsa)** | **Conserved-C or non-conserved-NC microRNA target site** | **Absence of/destroyed target site** | **Presence of/created target site** | **Difference in context+ score* (TargetScan)** |
| --- | --- | --- | --- | --- | --- | --- |
| rs11636419 | A | miR-1343, miR-6783-3p |  |  | X | -0.192, -0.195 |
| rs56141902 | G | miR-6746-5p, miR-6771-5p | NC | X |  | -0.135, -0.143 |
| “ | A | miR-1273h-5p, miR-149-3p, miR-30b-3p, miR-30c-1-3p, miR-30c-2-3p, miR-3689a-3p, miR-3689b-3p, miR-3689c, miR-4728-5p, miR-6779-5p, miR-6780a-5p, miR-6785-5p, miR-6788-5p, miR-6799-5p, miR-6883-5p, miR-7106-5p, miR-887-5p, miR-890 |  |  | X | -0.217, -0.086, -0.175, -0.042, -0.048, -0.185, -0.185, -0.185, -0.103, -0.185, -0.206, -0.076, -0.045, -0.106, -0.076, -0.083, -0.019, -0.016 |
| rs45599945 | A | miR-3934-5p |  |  | X | -0.107 |
| “ | G | miR-4755-3p | NC | X |  | -0.24 |
| rs28399502 | C | miR-548q, miR-582-3p | C | X |  | -0.197, -0.085 |
| “ | A | miR-1227-3p, miR-550b-2-5p |  |  | X | -0.134, -0.16 |
| rs35742808 | A | miR-4504 | NC | X |  | -0.172 |
| “ | G | miR-1273c, miR-4670-5p |  |  | X | -0.298, -0.221 |
| rs3181842 | C | miR-4537 |  |  | X | -0.394 |
| “ | T | miR-6807-5p | NC | X |  | -0.127 |
| rs138892132 | A | miR-3176, miR-3922-3p, miR-4672, miR-6768-5p | NC | X |  | -0.152, -0.18, -0.059, -0.026 |
| “ | T | miR-3190-5p, miR-3926, miR-548s |  |  | X | -0.158, -0.103, -0.098 |
| rs148726498 | T | miR-3622b-5p, miR-4645-5p, miR-4673 | NC | X |  | -0.093, -0.1, -0.156 |
| rs28969420 | G | miR-6512-3p, miR-661, miR-6720-5p, miR-6849-3p, miR-766-3p | C | X |  | -0.139, -0.194, -0.157, -0.224, -0.119 |
| “ | T | miR-4463, miR-4494 |  |  | X | -0.103, -0.098 |
| rs35462975 | T | miR-216b-5p, miR-4434, miR-4516, miR-4531, miR-5703 | C (miR-216b-5p)  NC | X |  | -0.049, -0.031, -0.027, -0.113, -0.034 |
| “ | C | miR-6894-5p, miR-7154-3p, miR-765, miR-766-5p |  |  | X | -0.234, -0.170, -0.084, -0.09 |
| rs28969421 | G | miR-3622a-3p, miR-3622b-3p, miR-6765-3p | C | X |  | -0.23, -0.209, -0.069 |
| “ | A | miR-8056 |  |  | X | -0.089 |
| rs1038376 | T | miR-3914 |  |  | X | -0.094 |
| rs707265 | G | miR-3622a-5p, miR-4269, miR-6715b-5p | NC | X |  | -0.313, -0.201, -0.192 |
| rs1042389 | T | miR-4476, miR-6077, miR-6876-5p, miR-8065 | C | X |  | -0.045, -0.104, -0.045, -0.093 |
| “ | C | miR-3612, miR-650, miR-4266, miR-4779, miR-4695-5p, miR-765 |  |  | X | -0.164, -0.164, -0.177, -0.09, -0.305, -0.155 |
| rs3732358 | G | miR-500b-3p, miR-597-5p, miR-6814-5p, miR-6879-3p | C (miR-500b-3p, miR-6814-5p)  NC | X |  | no change, -0.081, no change, no change |
| rs3732359 | G | miR-3136-3p, miR-7155-3p | C | X |  | -0.157, -0.157 |
| “ | A | miR-362-5p, miR-500b-5p, miR-501-5p |  |  | X | -0.128, -0.13, -0.008 |
| rs10511395 | C | miR-1254, miR-3116  miR-4254, miR-505-5p | C  NC (miR-505-5p) | X |  | -0.099, -0.08, -0.07, -0.129 |
| “ | A | miR-1225-3p, miR-1233-3p, miR-1245b-5p, miR-3142, miR-6807-5p |  |  | X | -0.135, -0.13, -0.119, -0.1, -0.081 |
| rs3732360 | C | miR-4763-5p, miR-4787-3p, miR-668-5p, miR-6720-3p | C | X |  | -0.153, -0.246, -0.214, -0.392 |
| “ | T | miR-500a-3p, miR-501-3p, miR-502-3p, miR-6804-3p |  |  | X | -0.107, -0.134, -0.134, -0.269 |
| rs73854723 | T | miR-4480 | C | X |  | -0.096 |
| rs1054190 | C | miR-1250-5p |  |  | X | -0.545 |
| “ | T | miR-148a-3p, miR-148b-3p, miR-152-3p, miR-361-5p, miR-374a-3p, miR-5094, miR-520f-3p | NC  C (miR-520f-3p) | X |  | -0.134, -0.134, -0.134, -0.019, -0.054, -0.195, -0.157 |
| rs1054191 | G | miR-371b-3p, miR-4258, miR-4707-3p | C (miR-371b-3p)  NC | X |  | -0.137, -0.247, -0.541 |
| “ | A | miR-1271-3p, miR-33b-3p, miR-4722-3p, miR-4763-5p,  miR-4769-3p, miR-515-3p, miR-519e-3p, miR-550a-3-5p,  miR-550a-5p, miR-6727-3p, miR-6817-5p, miR-6894-3p |  |  | X | -0.115, -0.087, -0.166, -0.433, -0.083, -0.078, -0.087, -0.106, -0.106, -0.166, -0.102, -0.146 |
| rs3814057 | A | miR-148b-5p, miR-2116-5p, miR-3168,  miR-5584-3p, miR-6800-5p, miR-6802-5p, miR-6874-3p | C | X |  | -0.074, -0.118,  -0.09, -0.246,  -0.133, -0.15,  -0.081 |
| rs3814058 | T | miR-489-3p, miR-514a-3p, miR-514b-3p | C | X |  | -0.117, -0.113, -0.113 |
| “ | C | miR-1256, miR-1271-5p, miR-182-5p, miR-4642, miR-96-5p, |  |  | X | -0.15, -0.155, -0.147, -0.17, -0.155 |
| rs45564134 | G | miR-3197, miR-3656, miR-4649-3p, miR-6087, miR-6765-5p | Ancestral allele is unknown |  |  | -0.155, -0.207, -0.044, -0.356,  -0.094 |
| “ | - | miR-1914-3p, miR-3184-5p, miR-423-5p, miR-4651, miR-5194,  miR-608, miR-6732-5p, miR-6734-5p, miR-6738-5p, miR-6752-5p, miR-6842-5p, miR-7110-5p |  |  |  | -0.127, -0.114,  -0.118, -0.23,  -0.123, -0.183,  -0.119, -0.12,  -0.12, -0.162,  -0.162, -0.181 |
| rs33972239 | T | miR-1305 | Ancestral allele is unknown |  |  | 0.026 |
| “ | - | miR-197-3p, miR-5096 |  |  |  | -0.083, -0.086 |

*Difference in context+ scores between the reference and derived alleles for each SNP, caused by a SNP in the microRNA target site, a more negative difference in context+ scores indicates an increased likelihood that the target sites are either absent or present due to the variant allele.
