# Supplementary material for: Genetic variation in the 3′-UTR of CYP1A2, CYP2B6, CYP2D6, CYP3A4, NR1I2, and UGT2B7: potential effects on regulation by microRNA and pharmacogenomics relevance
Source: Front Genet. 2014 Jun 4;5:167. doi: 10.3389/fgene.2014.00167 (PMC4044583; doi:10.3389/fgene.2014.00167)
Supplement: Supplementary file 1 [file DataSheet1.ZIP › 80478_Dandara_Data_Sheet_3.DOCX]

**Supplementary Table S3: Potential effect of novel genetic variation identified in the 3'-UTR on microRNA targeting**

| **Novel SNPs** | **Allele** | **MicroRNA ID (hsa)** | **Conserved-C or non-conserved-NC microRNA target site** | **Absence of/destroyed target site** | **Presence of/created target site** | **Maximum free binding energy* (DIANA-microT)** |
| --- | --- | --- | --- | --- | --- | --- |
| g.74755658G>A | G | miR-4755-3p | NC |  | X | -48.9 |
|  | A | miR-4453, miR-4538, miR-548s | NC |  | X | -42.9, -50.6, -40.2 |
| g.74756006G>A | G | miR-3150a-5p, miR-3150b-5p | NC |  | X | -48.2, -51.1 |
|  | A | miR-624 | NC |  | X | -38.0 |
| g.74756039T>A | T | miR-4534, miR-1273f, | NC |  | X | -41.1, -41.6 |
|  | A | miR-571 | NC |  | X | -44.9 |
| g.74756176G>A | G | miR-148a | C |  | X | -43.2 |
|  | A | miR-4302, miR-648 | NC |  | X | -42.1 |
| g.41016968T>A | T | miR-582-3p, miR-549 | NC |  | X | -40.8, -41.9 |
|  | A | miR-654-3p | NC |  | X | -45.0 |
| g.41017103C>T | C | miR-4252 | NC |  | X | -44.9 |
|  | T | miR-499a-3p | NC |  | X | -39.8 |
| g.41017242T>C | T | miR-128, miR-4672, miR-892a | C  NC  NC |  | X | -42.7, -46.0, -42.6 |
|  | C | miR-5096 | NC |  | X | -43.3 |
| g.41017290A>C | A | miR-1262, miR-4701-3p, miR-1224-3p | NC |  | X | -40.2, -44.7, -50.8 |
| g.41017450A>T | A | miR-3613-5p, miR-876-3p, miR-1273g | NC |  | X | -30.8, -37.4, -47.8 |
|  | T | miR-3165, miR-3691-5p | NC |  | X | -47.0, -49.2 |
| g.41017486C>T | T | miR-3613-3p, miR-371b-5p | NC |  | X | -41.7, -43.2 |
| g.41017525A>T | A | miR4257, miR-4724-5p, miR-4539 | NC |  | X | -42.8 -48.1, -53.6 |
|  | T | miR-140-3p | NC |  | X | -46.3 |
| g.41017749C>A | C | miR-3150a-3p |  |  | X | -51.0 |
|  | A | miR-150, miR-323b-3p | C  NC |  | X | -47.9, -46.6 |
| g.41017763C>A | C | miR-3123, miR-4311, miR-4753-3p | NC |  | X | -25.8, -36.0, -39.8 |
|  | A | miR-3121-3p | NC |  | X | -40.4 |
| g.41017847A>G | A | miR-3149, miR-4666-3p, miR-338-5p, miR-485-3p | NC |  | X | -39.5, -33.7, -43.5, -48.1 |
|  | G | miR-3145-3p | NC |  | X | -38.4 |
| g.99784127T>C | T | miR-3065-5p | NC |  | X | -43.3 |
|  | C | miR-4699-3p | NC |  | X | -39.1 |

*Maximum free binding energy as an indication of the predicted strength of microRNA targeting for each allele.
